# COVID-19 Vaccination Intention and Factors Associated with Hesitance and Resistance in the Deep South: Montgomery, Alabama

**DOI:** 10.3390/tropicalmed7110331

**Published:** 2022-10-25

**Authors:** Cicily A. Gray, Grace Lesser, Yuqi Guo, Swapn Shah, Shauntice Allen, Larrell L. Wilkinson, Omar T. Sims

**Affiliations:** 1 Department of Human Studies, Community Health and Human Services Program, School of Education, University of Alabama at Birmingham, Birmingham, AL 35294, USA; 2 School of Nursing, Yale University, New Haven, CT 06477, USA; 3 School of Social Work, College of Health and Human Services, University of North Carolina at Charlotte, Charlotte, NC 28223, USA; 4 School of Data Science, University of North Carolina at Charlotte, Charlotte, NC 28223, USA; 5 Department of Computer Science, University of North Carolina at Charlotte, Charlotte, NC 28223, USA; 6 Department of Environmental Health Sciences, School of Public Health, University of Alabama at Birmingham, Birmingham, AL 35294, USA; 7 Department of Social Work, College of Arts and Sciences, University of Alabama at Birmingham, Birmingham, AL 35294, USA; 8 Department of Health Behavior, School of Public Health, University of Alabama at Birmingham, Birmingham, AL 35294, USA; 9 Center for AIDS Research, School of Medicine, University of Alabama at Birmingham, Birmingham, AL 35294, USA; 10 Integrative Center for Aging Research, School of Medicine, University of Alabama at Birmingham, Birmingham, AL 35294, USA; 11 Center for Clinical and Translational Science, School of Medicine, University of Alabama at Birmingham, Birmingham, AL 35294, USA; 12 African American Studies, College of Arts and Sciences, University of Alabama at Birmingham, Birmingham, AL 35294, USA

**Keywords:** COVID-19, vaccine, acceptance, hesitance, resistance, the Deep South

## Abstract

Using COVID-19-related survey data collected from residents in the city of Montgomery, Alabama, this study assessed the prevalence of COVID-19 vaccine acceptance, hesitance, and resistance, and identified factors associated with COVID-19 vaccine hesitance and resistance. To analyze the survey data (n = 1000), a consolidation approach (machine learning modeling and multinomial logistic regression modeling) was used to identify predictors of COVID-19 vaccine hesitancy and resistance. The prevalence of vaccine acceptance, hesitancy, and resistance was 62%, 23%, and 15%, respectively. Female gender and a higher level of trust that friends and family will provide accurate information about the COVID-19 vaccine were positively associated with vaccine hesitancy. Female gender and higher trust that social media will provide accurate information about COVID-19 were positively associated with vaccine resistance. Factors positively associated with COVID-19 vaccine hesitance and resistance in the study’s geographical area are worrisome, especially given the high burden of chronic diseases and health disparities that exist in both Montgomery and the Deep South. More research is needed to elucidate COVID-19 vaccination attitudes and reasons for non-acceptance of the COVID-19 vaccine. Efforts to improve acceptance should remain a priority in this respective geographical area and across the general population.

## 1. Introduction

Coronavirus Disease 2019 (COVID-19) is caused by severe acute respiratory syndrome coronavirus-2 (SARS-CoV-2), it is easily transmitted person-to-person through respiratory droplets [1,2], and it can lead to illness and hospitalization [3]. Severe COVID-19 can cause lasting lung and heart damage, respiratory failure, kidney failure, and death [4,5]. During the early phases of the COVID-19 pandemic, the United States (U.S.) reported the highest excess all-cause mortality rate in the world (19.5/100,000) and is now leading the pandemic with approximately 91 million confirmed cases, approximately 5 million COVID-related hospitalizations, and over 1 million COVID-related deaths [6,7].

COVID-19 vaccination reduces the likelihood of viral transmission, hospitalization, illness severity, and mortality associated with COVID-19 [8]. Though all three COVID-19 vaccines (Pfizer-BioNTech, Moderna, and Janssen Biotech) have been demonstrated to be safe and highly effective (up to 93% efficacy), many people are hesitant towards accepting the vaccine (Self, 2021) (FDA, 2021). In turn, the World Health Organization (WHO) declared COVID-19 vaccine hesitance and resistance a top 10 global health threat [9]. For example, in the U.S. 79% of eligible adults and children have received only one vaccine dose, only 67% have been fully vaccinated, and 48% have received one dose of the vaccine booster [7]. The U.S. is currently ranked 65th globally in terms of the percentage of those who are fully vaccinated [10].

Of particular concern is COVID-19 vaccination rates in the Deep South states such as Alabama, Louisiana, Mississippi, Georgia, and South Carolina, whose rates are trending below national vaccination rates. Currently, only 64% of the eligible population in the aforementioned Deep South states have received at least one dose, while only 54% have received two doses [11]. Lower COVID-19 vaccination rates in the Deep South are certainly cause for concern given the higher prevalence of chronic illness, and a higher concentration of African Americans (60%) when compared to other states [12,13,14].

Of the Deep South states, Alabama has reported 1.4 million COVID-19 cases and has the 8th highest case fatality rate (1.38) in the country [11]. Yet, only 60% of eligible Alabama residents have received one dose, only 50% have been fully vaccinated, and only 18% have received an additional dose of the COVID-19 vaccine [15]. Montgomery County is mostly populated by the City of Montgomery (Alabama’s state capitol) which has a large population of African Americans (60%) [16]. It should be noted COVID-19 vaccination rates in Montgomery County are slightly below the state average. Approximately 58% of Montgomery County residents received at least one vaccine dose and approximately 46% have received a second dose [15].

Increased vaccine acceptance is important in mitigating individual health, community health, and public health consequences of COVID-19, particularly in efforts to protect highly susceptible, at-risk populations (older adults and people of color) who tend to experience increased risk of exposure, more severe illness, and higher mortality rates due to pre-existing co-morbidities and other related socioeconomic factors [7,17,18]. Though several studies have published on recommendations to improve COVID-19 vaccine rates and on COVID-19 vaccine intention [19,20,21,22,23,24], more research is needed to explore COVID-19 vaccine intentions in the Deep South, especially given the low COVID-19 vaccine rates in the Deep South. Empirical data would be advantageous for use among community and public health professionals in the Deep South, who are engaged in health education and health promotion efforts to curve or reduce COVID-19 hesitance and resistance. Hence, the objectives of this study were to assess the prevalence of COVID-19 vaccine acceptance, hesitancy, and resistance, and to identify factors associated with COVID-19 vaccine hesitancy and resistance. To achieve study objectives, we conducted a secondary analysis of COVID-19-related survey data collected from residents in the city of Montgomery, Alabama.

## 2. Materials and Methods

We conducted a secondary analysis of survey data collected by Consensus Strategies for Partners in Health (PIH-USA). The survey was originally conducted to inform a COVID-19 vaccine promotion campaign in Montgomery, Alabama spearheaded by PIH-USA. To obtain a representative sample of participants from the city of Montgomery, Consensus Strategies used a random stratified probabilistic sampling strategy to identify and recruit participants from a database of telephone numbers of Montgomery residents (>18 years old) for survey participation. The survey was administered using both an automated telephone interviewing (ATI) system and short message service (SMS). A total of 1000 participants completed the survey (ATI, n = 386; SMS, n = 614) between 22 January 2021 and 24 January 2021. Data collected through ATI and SMS were entered directly into a computerized database through the numbers selected by the interviewee while responding to survey questions. The margin of sampling error for the sample was +/−3% in 19 of 20 cases, which was a small sampling error for a probability-based survey where participants have a known and non-zero change of being included in the sample. The data were rake weighted by gender, age, race, and education based on 2019 American Community Survey 5-year estimates. The institutional review board (IRB) at the University of Alabama at Birmingham approved secondary analysis of the de-identified dataset.

### 2.1. Outcomes of Interest

The main outcome was COVID-19 vaccination intention. Outcomes of interest were: (1) the prevalence of COVID-19 vaccine acceptance, hesitancy, and resistance, (2) and factors associated with COVID-19 vaccine hesitancy and resistance. The following question was used to categorize patients into COVID-19 vaccination intention, After the COVID-19 vaccine becomes available to you, when, if at all, do you plan to get it (as soon as it’s available; a few weeks after; a few months after; a year or more after it’s available; I won’t get the vaccine ever)? Participants who endorsed as soon as it’s available to them were defined as being accepting of the COVID-19 vaccine (i.e., COVID-19 acceptance), those who endorsed I won’t get the vaccine ever were defined as being resistant to the COVID-19 vaccine (i.e., COVID-19 vaccine resistance), and those who endorsed a few weeks, a few months, and a year or more after it’s available to them were defined as being hesitant to the COVID-19 vaccine (i.e., COVID-19 vaccine hesitancy). In summary, COVID-19 vaccine acceptance was defined as intentions to be vaccinated as soon as the vaccine was available, hesitance was defined as intentions of waiting a few weeks, a few months, or longer after availability to get vaccinated, and resistance was defined as no intentions of getting vaccinated.

### 2.2. Independent Variables

#### 2.2.1. Demographics

Demographics included age, race, gender, highest level of education completed, and if children <18 years old were living at home.

#### 2.2.2. COVID-19 Positivity and Mask Wearing 

Participants reported if they or anyone they knew tested positive for COVID-19 and if they knew someone who had received the COVID-19 vaccine; and reported the frequency of wearing a mask while in public places (1 = never; 2 = rarely; sometimes; 3 = most of the time; 4 = all of the time).

#### 2.2.3. COVID-19 Information and Messaging 

The following two survey questions asked questions related to COVID-19 information and messaging: What information about COVID-19 has been the most difficult for you to understand or find (how to keep yourself safe from COVID-19; when and were to get tested for COVID-19; what to do when you feel sick; information about COVID-19 vaccine safety; information about COVID-19 vaccine availability); The public health messages I have heard about COVID-19 have been clear and easy to understand (1 = completely disagree; 2 = somewhat disagree; 3 = somewhat agree; 4 = completely agree).

#### 2.2.4. Level of Trust in the Accuracy of Information about the COVID-19 Vaccine from Sources

Participants were asked to rate their level of trust (1 = do not trust at all; 2 = somewhat distrust; 3 = neutral; 4 = somewhat trust; 5 = completely trust) that the following sources would provide them with accurate information about the COVID-19 vaccine: employers, healthcare providers, locally elected government officials, elected officials in the federal government, officials in the state’s department of public health, family and friends, local television news, national television news, social media (e.g., Facebook, Twitter, and Instagram), and religious organizations.

#### 2.2.5. COVID-19 Vaccine Protection, Vaccine Development, and Vaccine Side-Effects

Participants were asked about their level of confidence in the COVID-19 vaccine being able to protect them and their family from getting sick from COVID-19 and level of confidence that the development of a COVID-19 vaccine is taking the needs of Black people into account (1 = don’t know; 2 = not at all confident; 3 = not too confident; 4 = somewhat confident; 5 = very confident). The following question assessed side-effect concerns, How concerned are you that there would be side-effects from the COVID-19 vaccine (1 = don’t know; 2 = not at all concerned; 3 = not too concerned; 4 = somewhat concerned; 5 = very concerned)?

#### 2.2.6. Racism in Healthcare

The following survey question asked how often they thought the healthcare system treated people unfairly based on their race and ethnic background, Generally speaking, how often do you think our healthcare system treats people unfairly based on their race or ethnic background (1 = never; 2 = rarely; 3 = sometimes; 4 = most of the time; 5 = all of the time)?

#### 2.2.7. Food and Financial Impacts from the COVID-19 Pandemic

Participants were asked if the COVID-19 pandemic had caused a lack of food at any time, and if their household were *better off or worse off* than they were before the pandemic, and if they thought during the next 12 months that they and their household would be better off, worse off, or about the same as now.

#### 2.2.8. Mandatory COVID-19 Vaccinations

The following survey question asked about mandatory vaccinations, Though there are no plans for it, do you feel making the COVID-19 mandatory statewide is a beneficial or harmful idea (beneficial; harmful; neither; don’t know)?

### 2.3. Statistical Analysis

**Figure 1:**
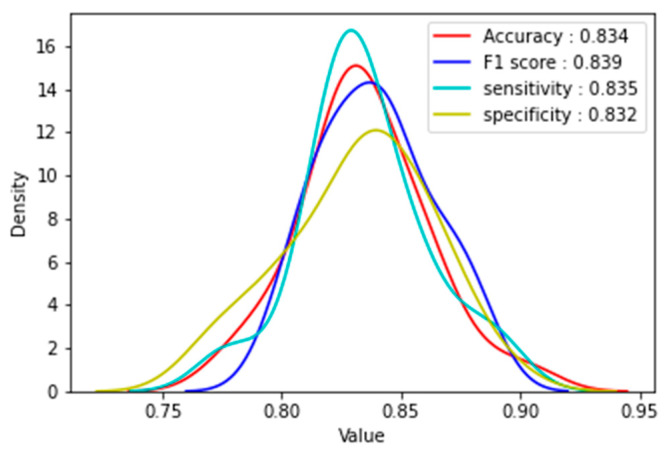
Performance of the Random Forest Model.

Measures of central tendency and frequency distributions were used to characterize the sample. To robustly identify and analyze factors associated with COVID-19 vaccination intention, we adopted a consolidation approach that combines data-driven models (machine learning models) and hypothesis-driven models (regression models) [25,26,27,28,29,30]. The random forest (RF) model was developed to identify important factors that can predict individuals’ COVID-19 vaccination intention. RF model is a machine learning method for classification and regression tasks that operates by constructing a multitude of decision trees (by combining and averaging more than 100 decision tree models) at training time. Important factors associated with COVID-19 vaccination intention were selected by most decision trees. In this study, the data was split into a training set (800/1000, 80%) to develop RF models and a testing set (200/1000, 20%) to validate the performance of RF models. To ensure the rigor of prediction, we balanced the data with the in-built parameter “class_weight” of random forest classifier by setting it to “balanced” which helps us optimize the scoring for the minority class by assigning weights to the classes. Class weights were calculated as weight(i) = n_samples/(n_classes ∗ n_samples(i). Grid search with a 10-fold cross validation method was used to tune and adjust the hyperparameters in the RF model. The performance of the RF model was evaluated by testing the accuracy (0.83), F1 score (0.84), sensitivity (0.84), and specificity (0.83) (Figure 1). Based on Gini impurities of the features, a list ranking and scoring variables with important features of predicting the outcome, COVID-19 vaccination intention, was generated. Multinomial logistic regression was used to identify predictors of COVID-19 vaccine hesitancy and resistance. Python 3.7.6 and RStudio 1.3.1056 were used to develop the RF model, and Stata 12 was used to run multinomial logistic regression.

## 3. Results

### 3.1. Demographics

Nearly one-third (31%) of participants were 45–64 years old, and the majority were Black/African American (61%) and female (52%) (Table 1). More than one-third had an undergraduate or post-graduate degree (35%), and most did not have children <18 years old living at home (70%). 

### 3.2. COVID-19 Positivity and Mask Wearing

The majority of participants knew someone who tested positive for COVID-19 (68%), and only a few had ever tested positive for COVID-19 (5%). The majority knew someone who had received the COVID-19 vaccine (61%). The mean (standard deviation) rate of mask wearing in public spaces was 4.6 (0.85) (i.e., all of the time). 

### 3.3. COVID-19 Information and Messaging

Participants reported that information about COVID-19 vaccine availability (40%) was most difficult for them to understand and to find, followed by when and where to get tested for COVID-19 (30%) and information about COVID-19 vaccine safety (16%). The mean level of agreement that public health messages they had heard about COVID-19 were clear and easy to understand was 3.1 (0.9) (i.e., somewhat agree).

### 3.4. Level of Trust in the Accuracy of Information about the COVID-19 Vaccine from Sources

Participants’ mean level of trust that the following sources would provide accurate information about the COVID-19 vaccine was as follows: employer, 3.4 (1.3) (i.e., neutral); healthcare providers, 4.1 (1.1) (i.e., somewhat trust); locally elected government officials, 3.2 (1.3) (i.e., neutral); elected officials in the federal government, 3.2 (1.4) (i.e., neutral); officials in the state’s department of public health, 3.7 (1.2) (i.e., somewhat trust); family and friends, 3.7 (1.1) (i.e., somewhat trust); local television news 3.5 (1.2) (i.e., somewhat trust); national television news, 3.2 (1.3) (i.e., neutral); social media, 2.3 (1.2) (i.e., somewhat distrust); religious organizations, 3.2 (1.3) (i.e., neutral). 

### 3.5. COVID-19 Vaccine Protection, Vaccine Development, and Vaccine Side-Effects

The mean level of confidence that the vaccine would protect them and their family from getting sick with COVID-19 was 3.7 (1.2) (i.e., somewhat confident), the mean level of confidence that the development of a COVID-19 vaccine is taking the needs of Black people into account was 3.4 (1.3) (i.e., not too confident), and the mean level of concern that there would be side-effects from the COVID-19 vaccine was 3.9 (1.2) (i.e., somewhat concerned).

### 3.6. Racism in Healthcare

Participants’ mean rate of the healthcare system treating people unfairly based on their race or ethnic background was 3.3 (1.2) (i.e., sometimes).

### 3.7. Food and Financial Impacts of the COVID-19 Pandemic and Mandatory Vaccinations 

More than one-quarter (29%) of participants reported that the COVID-19 pandemic had caused a lack of food. The majority reported that their household was worse off financially than they were before the pandemic (62%), and a minority (25%) thought during the next 12 months that they and their household would be worse off financially. Though there were no plans of a statewide COVID-19 vaccination mandate, half of participants (50%) thought it would be beneficial.

### 3.8. COVID-19 Vaccination Intention

The prevalence of vaccine acceptance, hesitancy, and resistance was 62%, 23%, and 15%, respectively. Of those with vaccine acceptance, the most frequent motivation for getting the vaccine right away was to protect self from COVID-19 (51%), followed by to help end the pandemic more quickly (21%) and to protect those around them from COVID-19 (17%); and the majority preferred to get vaccinated at their doctor’s office (33%), followed by the local the pharmacy (e.g., CVS or Walgreens) (29%). Of those with vaccine hesitancy, the most frequent reason for the wait was let high-risk people go first (47%), followed by wait until it is easier to get a COVID-19 vaccine (25%) and see how it works in other people (15%).

### 3.9. Feature Importance Analysis

To improve the performance of the multinomial regression model, only the top 15 variables with the highest scores and any demographic variables that were not in the top 15 (gender and race), a total of 17, from the feature importance analysis were entered into the multinomial logistic regression model. A detailed ranking of all variables from the feature importance analysis can be found in Table 2, but a few examples were: level of confidence in the COVID-19 vaccine providing protection from COVID-19; level of trust in accuracy of COVID-19 vaccine information from healthcare providers; frequency of mask wearing in public places, level of COVID-19 vaccine side-effects concerns; and level of trust in accuracy of COVID-19 vaccine information from locally elected government officials.

### 3.10. Multivariate Analysis: COVID-19 Vaccine Hesitancy

Female gender (adjusted odds ratio [aOR] = 1.95, confidence interval [CI]: 1.02–3.73) and a higher level of trust that friends and family will provide accurate information about the COVID-19 vaccine (aOR = 1.15, CI: 1.03–2.20) were positively associated with vaccine hesitancy (Table 3). Higher age (aOR = 0.42, CI: 0.29–0.61), a higher frequency of wearing masks in public places (aOR = 0.40, CI: 0.29–0.56), a higher level of confidence that the COVID-19 vaccine would protect self and family from getting sick from COVID-19 (aOR = 0.68, CI: 0.47–0.98), and clearer and easier public health messages about COVID-19 (aOR = 0.57, CI: 0.42–0.78) were negatively associated with vaccine hesitancy.

### 3.11. Multivariate Analysis: COVID-19 Vaccine Resistance

Female gender (aOR = 4.45, CI: 1.15–1.73) and higher trust that social media will provide accurate information about COVID-19 (aOR = 1.62, CI: 1.03–2.54) were positively associated with vaccine resistance. Higher age (aOR = 0.15, CI: 0.07–0.32), a higher frequency of wearing masks in public places (aOR = 0.26, CI: 0.14–0.47) and a higher level of confidence that the COVID-19 vaccine would protect self and family from getting sick from COVID-19 (aOR = 0.25, CI: 0.16–0.41) were negatively associated with vaccine resistance; and a higher level of trust that healthcare providers (aOR = 0.32, CI: 0.18–0.58), locally elected government officials (aOR = 0.55, CI: 0.32–0.94), and officials in the state’s department of public health (aOR = 0.53, CI: 0.30–0.93) will provide accurate information about the COVID-19 vaccine were negatively associated with vaccine resistance.

## 4. Discussion

Using COVID-19-related survey data collected from residents in the city of Montgomery, Alabama, this study assessed the prevalence of COVID-19 vaccine acceptance, hesitance, and resistance, and identified factors associated with COVID-19 vaccine hesitance and resistance. Several main findings emerged from the study. First, the prevalence of COVID-19 vaccine acceptance, hesitancy, and resistance was 62%, 23%, and 15%, respectively. Second, participants of female gender were more likely to be hesitant about the COVID-19 vaccine, and participants with a high level of trust that friends and family will provide accurate information about COVID-19 were more likely to be hesitant about the COVID-19 vaccine. High frequency of mask wearing, confidence in COVID-19 vaccination protection, and clear and easy to understand public health messages reduced the odds of COVID-19 vaccine hesitance. Third, female participants were more likely to be resistant to the COVID-19 vaccine, and those with high trust that social media will provide accurate information about COVID-19 were more likely to be resistant to the COVID-19 vaccine. However, high frequency of mask wearing and high confidence in COVID-19 vaccination protection reduced the odds of COVID-19 vaccine resistance; and trust that healthcare providers and local and federally elected government officials would provide accurate information about the COVID-19 vaccine reduced the odds of COVID-19 vaccine resistance.

A high proportion of participants in this study (38%) were hesitant or resistant about the COVID-19 vaccine. Similar studies conducted elsewhere have also reported high proportions of COVID-19 vaccine hesitance and resistance among adults [18,31,32,33]. High frequencies of COVID-19 vaccine hesitance and resistance in the study’s geographical area are worrisome, especially given the high burden of chronic diseases and health disparities (e.g., cardiovascular disease, diabetes, cancer, obesity) that exist in both Montgomery and the Deep South that are associated risk factors for COVID-19 infection. Wide non-willingness to accept the COVID-19 vaccine at the local level inhibits achievement of immunity at the population level. Those who are not vaccinated are not only at greater risk of acquiring COVID-19 compared to those who are vaccinated, but their risk for reinfection is more than two times higher than those who acquired COVID-19 and got vaccinated [34]. Altogether, the finding highlights a continued need for COVID-19 vaccination health education and health promotion efforts and campaigns to improve COVID-19 vaccination acceptance.

Though the current study did not ascertain why participants of female gender were more likely to have COVID-19 vaccine resistance and hesitance than participants of male gender, findings from several other studies have also demonstrated that females are more likely than males to be resistant or hesitant about the COVID-19 vaccine [24,32,35,36,37]. Similarly, those respective studies did not ascertain why females are more likely to have COVID-19 vaccine resistance and hesitance. In general, it has been noted that females more frequently express concerns about the safety of vaccines and a lack of trust in the quality and impartiality of information provided by healthcare professionals [24]. However, qualitative studies seeking to give voice to and examine concerns that females have about the COVID-19 vaccine are limited, and more qualitative studies are needed to fill this knowledge gap.

In terms of the finding that participants with high trust that social media will provide accurate information about COVID-19 had increased odds of COVID-19 vaccine resistance, it is probable that considerable amounts of misinformation widely available on social media platforms stemming from anti-vaccine efforts may be an underlying contributing factor of COVID-19 vaccine resistance [38]. It has been noted that social medial platforms have been purposefully used as “echo chambers” to circulate misinformation from unreliable or unverified sources about COVID-19 leading to COVID-19 vaccine resistance [39]. The examination of COVID-19 vaccine content on social media determined nearly 23% of content shared on social media was misleading, and that fact-based content from government health agencies was underrepresented. To combat misinformation shared on social media platforms, community health professionals and public officials are encouraged to regularly use social media to expeditiously disseminate relevant, timely, and empirically based COVID-19 information [40].

Findings also demonstrate the role and importance of COVID-19 vaccine confidence, trust in health care providers and public officials, and the frequency of mask wearing as deterrents of COVID-19 vaccine hesitance and resistance. As such, community health and public health professionals are encouraged to continue to sustain current health education and promotion efforts in these respective education-related domains about COVID-19 and the COVID-19 vaccine. Dissemination of evidence-based COVID-19 education, ideally grounded in elements of health behavior theory (e.g., the health belief model [HBM] or the Theory of Planned Behavior [TBP]), has the potential to address and target attitudes, thoughts, perceptions, and social influences that reinforce non-acceptance of the COVID-19 vaccine [18,41,42].

This study had some noteworthy limitations as well as strengths. The survey data was cross-sectional, the participant non-response rate was not available, and the survey data did not longitudinally collect data over time on potential changes in participants’ COVID-19 vaccination intention. The analysis heavily relied on self-reported survey data, which increases the chances of under or overreporting by participants. Participants were recruited from a single city limiting generalizability to cities with similar sociodemographic characteristics as Montgomery, and the sample was not homogeneous. However, the use of a random stratified probabilistic sampling strategy facilitated recruitment of a representative sample of the city of Montgomery, and the data was rake weighted. African Americans constituted the majority (61%) rather than the minority of the sample. Machine learning was employed to identify the most important independent variables for inclusion in the multinomial logistic regression.

## 5. Conclusions

It still remains clear why the WHO declared COVID-19 vaccine hesitance and resistance a top 10 global health threat. Intentions for COVID-19 vaccination uptake are suboptimal, and efforts to improve acceptance should remain a priority at the community level and across the general population. Mask wearing, vaccine confidence, clear and easy to understand public health messages, and public trust in health care providers and officials as sources for accurate COVID-19 information appear to deter COVID-19 vaccine hesitance and resistance. More research is needed to elucidate COVID-19 vaccination attitudes and reasons for non-acceptance of the COVID-19 vaccine among people with reduced odds of uptake. If findings from those respective studies are used to develop novel health education and promotion intervention approaches or are used to enhance existing approaches and strategies, it is plausible that in the near future that COVID-19 vaccine hesitance and resistance could be dislodged as a barrier to effective public health management of the COVID-19 pandemic.

## Figures and Tables

**Table 1:** Participant Characteristics.

Variables	n (%)/Mean (sd)
N = 1000	
**Demographics**	
Age	
18–29	216 (21.6%)
30–44	270 (27.0%)
45–64	311 (31.1%)
≥65	203 (20.3%)
Race	
Black/African American	607 (60.7%)
White/Caucasian	304 (30.4%)
Other	89 (8.9%)
Gender	
Male	463 (46.3%)
Female	523 (52.3%)
Prefer to self-describe	14 (1.4%)
Highest Level of Education Completed	
High school graduate or less	350 (35.0%)
Some college/technical school	300 (30.0%)
University undergraduate degree	200 (20.0%)
Post-graduate degree	150 (15.0%)
Have children <18 years old living in your house	296 (29.6%)
**COVID-19 Positivity and Mask Wearing**	
Have you or anyone you know tested positive for COVID-19?	
Yes, I have	54 (5.4%)
Yes, someone I know	683 (68.3%)
Yes, I have and someone I know	37 (3.7%)
No, neither	225 (22.5%)
Do you know anyone who has received a COVID-19 vaccine shot?	
No	386 (38.6%)
Yes	614 (61.4%)
Since the start of the new year, how often have you worn a mask while in public places?	
Never	20 (2.0%)
Rarely	25 (2.5%)
Sometimes	56 (5.6%)
Most of the time	134 (13.4%)
All of the time	766 (76.6%)
**COVID-19 Information and Messaging**	
From the list below, what information about COVID-19 has been the	
most difficult for you to understand or find?	
How to keep yourself safe from COVID-19	152 (10.56%)
When and where to get tested for COVID-19	242 (24.90%)
What to do when you feel sick	75 (8.33%)
Information about COVID-19 vaccine safety	220 (16.18%)
Information about COVID-19 vaccine availability	312 (40.01%)
The public health messages I have heard about COVID-19 have been clear and easy to understand	3.1 (0.9)
Trust	
To what extent do you trust each of the following sources to provide you with accurate information about the COVID-19 vaccine:	
Employer	3.4 (1.3)
Healthcare providers	4.1 (1.1)
Locally elected government officials	3.2 (1.3)
Elected officials in the federal government	3.2 (1.4)
Officials in the state’s department of public health	3.7 (1.2)
Friends and Family	3.7 (1.1)
Local television news	3.5 (1.2)
National television news	3.2 (1.3)
Social media, such as Facebook, Twitter and Instagram	2.3 (1.2)
Religious organizations	3.2 (1.3)
**COVID-19 Vaccine Protection, Vaccine Development, and Vaccine Side-Effects**	
Based on what you know about the COVID-19 vaccine, how confident would you be that it would protect you and your family from getting sick with COVID-19?	3.7 (1.2)
How confident are you that the development of the COVID-19 vaccine is taking the needs of Black people into account?	3.4 (1.3)
How concerned are you that there would be side-effects from the new COVID-19 vaccines?	3.9 (1.2)
Racism in Healthcare	
Generally speaking, how often do you think our healthcare systemtreats people unfairly based on their race or ethnic background?	3.3 (1.2)
Food and Financial Impacts of the COVID-19 Pandemic and Mandatory Vaccinations	
Has the COVID-19 pandemic caused you to have a lack of food at any time?	
No	714 (71.4%)
Yes	286 (28.6%)
Since the start of the COVID-19 pandemic, would you say you and your household are better off or worse off financially than you were before the pandemic?	
Better off	380 (38.0%)
Worse off	620 (62.0%)
Now looking ahead, do you think during the next 12 months you and your household will be better off financially or worse off, or just about the same as now?	
Better off	181 (18.1%)
Worse off	250 (25.0%)
About the same	569 (56.9%)
Though there are no plans for it, do you feel making the COVID-19 vaccine mandatory statewide is a beneficial or harmful idea?	
Don’t know	159 (15.9%)
Neither	93 (9.3%)
Harmful	247 (24.7%)
Beneficial	500 (50.0%)
**Vaccination Intention**	
Vaccine Intention when the Vaccine Becomes Available to You	
Yes/Acceptance (as soon as it’s available)	623 (62.3%)
Wait/Hesitancy (combine a few weeks/months/a year after it’s available)	226 (22.6%)
Resistance/No (I won’t get the vaccine ever)	151 (15.1%)
Of those with vaccine acceptance, the main motivation to get the vaccine right away	
To protect myself from COVID-19	318 (51.0%)
I want to protect my community	55 (8.9%)
To protect those around me from COVID-19	104 (16.8%)
To help end the pandemic more quickly	126 (20.3%)
Other	19 (3.0%)
Of those with vaccine acceptance, where would you prefer to get vaccinated	
Local Pharmacy like CVS or Walgreens	254 (28.9%)
Hospital	169 (19.2%)
Sports Stadium	18 (2.0%)
Your Doctor’s Office	289 (32.9%)
Mobile unit deployed by the department of health in your neighborhood	87 (9.9%)
Local schools	17 (1.9%)
At a mall	16 (1.8%)
Somewhere else	29 (3.3%)
Of those with vaccine hesitancy, the top reason for the wait	
See how it works in other people	26 (15.1%)
Let high-risk people go first	81 (47.2%)
Wait until it is easier to get one	43 (24.9%)
Other	22 (12.8%)

**Table 2:** Feature Importance Analysis: Ranking of Variables with Important Features of Predicting COVID-19 Vaccination Intention.

Importance Ranking	Feature Importance Scores	Variables
1	0.13	Level of confidence in the COVID-19 vaccine providing protection from COVID-19
2	0.09	Level of trust in accuracy of COVID-19 vaccine information: Healthcare providers
3	0.06	Frequency of mask wearing while in public places
4	0.06	Level of COVID-19 vaccine side-effects concerns
5	0.06	Level of trust in accuracy of COVID-19 vaccine information: Locally elected government officials
6	0.05	Age
7	0.04	Level of trust in accuracy of COVID-19 vaccine information: Officials in the state’s department of public health
8	0.04	Level of trust in accuracy of COVID-19 vaccine information: Local television news
9	0.04	Level of trust in accuracy of COVID-19 vaccine information: Elected officials in the federal government
10	0.03	Frequency of racism in healthcare system
11	0.03	Level of trust in accuracy of COVID-19 vaccine information: Employer
12	0.03	Public health messages: Clear and easy to understand
13	0.03	Level of trust in accuracy of COVID-19 vaccine information: Social media
14	0.03	Education level
15	0.03	Level of trust in accuracy of COVID-19 vaccine information: Family and friends
16	0.03	Level of trust in accuracy of COVID-19 vaccine information: Religious organizations
17	0.02	Level of trust in accuracy of COVID-19 vaccine information: National television news
18	0.02	Race
19	0.02	COVID-19 vaccine development is taking the needs of Black people into account
20	0.02	COVID-19 pandemic caused a lack of food at any time
21	0.02	COVID-19 positivity: You or anyone you know
22	0.02	Children <18 years old living at home
23	0.02	Future financial impact of the COVID-19 pandemic
24	0.02	Know anyone who has received the COVID-19 vaccine
25	0.01	Current financial impact of the COVID-19 pandemic
26	0.01	Gender

**Table 3:** Multinomial Regression Predicting COVID-19 Vaccination Intention.

	COVID-19 Vaccination Intention							
	Hesitancy (Reference: Acceptance)				Resistance (Reference: Acceptance)			
	aOR	95%, CI		*p*-Value	aOR	95%, CI		*p*-Value
Age	0.42	0.29	0.61	0.00	0.15	0.07	0.32	0.00
Race (reference: White/Caucasian)								
Black/African American	1.14	0.58	2.24	0.71	1.09	0.35	3.42	0.88
Other	1.96	0.60	6.43	0.27	2.25	0.99	2.11	0.05
Female (reference: male)	1.95	1.02	3.73	0.04	4.45	1.15	1.73	0.03
Education level	1.21	0.88	1.65	0.25	0.82	0.45	1.48	0.51
Frequency of wearing a mask while in public places	0.40	0.29	0.56	0.00	0.26	0.14	0.47	0.00
Based on what you know about the COVID-19 vaccine, how confident would you be that it would protect you and your family from getting sick with COVID-19?	0.68	0.47	0.98	0.04	0.25	0.16	0.41	0.00
How concerned are you that there would be side-effects from the COVID-19 vaccine.	0.57	0.32	1.04	0.07	0.78	0.48	1.27	0.31
Generally speaking, how often do you think our healthcare system treats people unfairly based on their race or ethnic background (i.e., racism in healthcare)	1.06	0.84	1.34	0.61	0.81	0.57	1.14	0.23
Public health messages I have heard about COVID-19 have been clear and easy to understand	0.57	0.42	0.78	0.00	1.24	0.67	2.30	0.49
To what extent do you trust that the following sources to provide you with accurate information about the COVID-19 vaccine:								
Healthcare providers	0.92	0.66	1.27	0.60	0.32	0.18	0.58	0.00
Locally elected government officials	0.84	0.57	1.24	0.37	0.55	0.32	0.94	0.03
Officials in the state’s department of public health	0.92	0.68	1.24	0.58	0.53	0.30	0.93	0.03
Local television news	0.93	0.65	1.34	0.70	0.78	0.50	1.21	0.27
Elected officials in the federal government	0.93	0.63	1.36	0.70	0.95	0.57	1.56	0.83
Employer	0.87	0.66	1.15	0.33	1.00	0.67	1.49	0.99
Social media, such as Facebook, Twitter, and Instagram	1.21	0.87	1.68	0.26	1.62	1.03	2.54	0.04
Friends and family	1.51	1.03	2.20	0.03	1.18	0.66	2.13	0.58

aOR = adjusted odds ration; CI = confidence interval.

## Data Availability

Data sharing is not applicable to this article.
